# Association of T Stage and Serum CEA Levels in Determining Survival of Rectal Cancer

**DOI:** 10.3389/fmed.2019.00270

**Published:** 2020-01-10

**Authors:** Shengwen Wu, Wenlong Gu

**Affiliations:** ^1^Department of General Surgery, The Affiliated Jianhu Hospital of Nantong University, Jianhu People's Hospital, Jianhu, China; ^2^Department of Medical Oncology, The Affiliated Jianhu Hospital of Nantong University, Jianhu People's Hospital, Jianhu, China

**Keywords:** T stage, carcinoembryonic antigen, rectal cancer, survival, SEER

## Abstract

**Purpose:** To investigate the association of T stage and serum carcinoembryonic antigen (CEA) levels in determining oncologic outcomes of rectal cancer.

**Methods:** Patients diagnosed with stage I–II rectal cancer patients were identified from the Surveillance, Epidemiology, and End Results database.

**Results:** In stage T1N0M0 disease, elevated level of serum CEA (C1) was associated with 227.6% increased risk of mortality compared to normal level of serum CEA (C0; hazard ratio = 3.276, 95% confidence interval = 2.781–3.858, *P* < 0.001).

**Conclusions:** Stage T1N0M0 rectal cancer, when involved in preoperative serum CEA elevation, may be a surrogate of biologically aggressive disease and correlate with unfavorable oncologic outcomes. Moreover, this subgroup of rectal cancer deserves more clinical attention of oncologists.

## Introduction

Rectal cancer is one of the most common malignancies worldwide (1). According to the American Joint Committee on Cancer (AJCC) staging system, the anatomic extent of primary tumor (T stage) is one of the most important prognostic predictors. However, patients with the same T stage of rectal cancer may show considerably different clinical outcomes.

First reported in 1965, carcinoembryonic antigen (CEA) is a 180–200 kDa glycoprotein and a member of the immunoglobulin superfamily (2). CEA is secreted by a variety of solid tumors, including 90% of colorectal cancers (3). As the single most important and reliable serum prognostic biomarker in colorectal cancer, elevated preoperative CEA levels are found to be associated with worse prognosis of colorectal cancer (4–9).

Previous studies have demonstrated that CEA was associated with cancer cell adhesion and innate immunity in colorectal cancer. In addition, CEA was also reported to facilitate attachment of colorectal cancer cells to sites of metastasis and support tumor progression (10–12).

Traditionally, the distant spread of tumor cells has been considered a late event, yet findings of several previous studies indicated that acquisition of metastatic potential could occur in the very early stage of tumor progression (13–16). Wo et al. (17) reported that a very small tumor size involved in lymph node positivity may be a surrogate for aggressive biology. We then suspect that very early stage rectal cancer with serum CEA elevation could suggest the early acquisition of metastatic potential and predict a very poor survival of rectal cancer.

However, to our knowledge, few studies were reported to investigate the association of T stage and serum CEA levels (C0 and C1) in determining prognosis of rectal cancer. Therefore, we conduct this large population-based study to examine whether very early T stage in the context of serum CEA elevation may be a surrogate for biologically aggressive disease and predict for poor cause-specific survival (CSS) of rectal cancer. To remove the effect of lymph node positivity on our research, we then excluded node-positive patients and focused the analysis on stage I–II patients.

## Patients and Methods

### Patient Selection in the Surveillance, Epidemiology, and End Results Database

**Figure 1:**
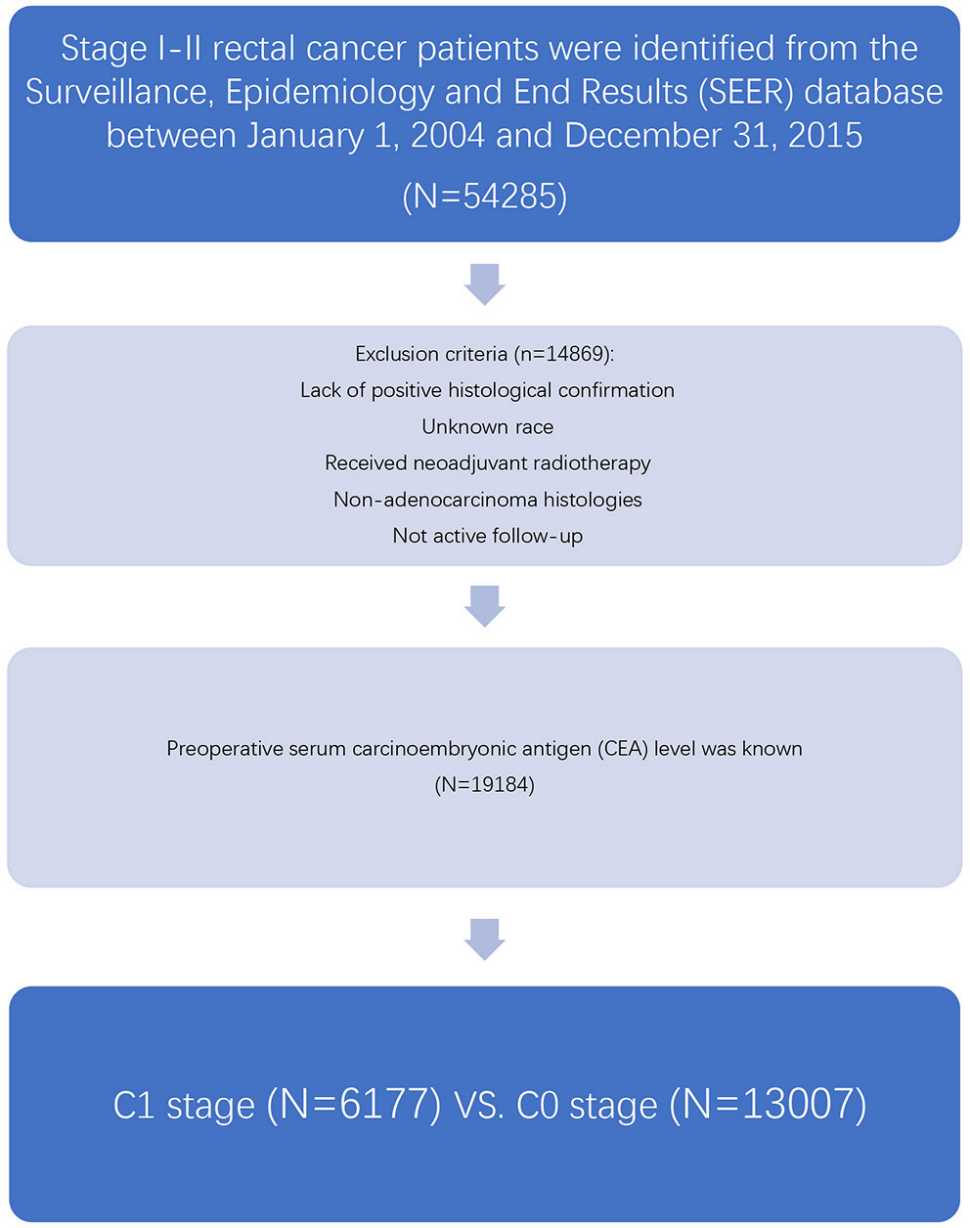
Flow chart of patient cohort definitions.

As an authoritative source of information on cancer incidence and survival in the USA and a comprehensive source of population-based information including all the newly diagnosed cancer cases occurring in Surveillance, Epidemiology, and End Results (SEER)-participating areas, the SEER database encompasses ~28% of the American population. The SEER database did not contain any identifiers and was publicly available for researchers. SEER^*^Stat is a software provided by the SEER program to obtain patient information using online access. At first, the case-listing session of the SEER^*^Stat software (SEER^*^Stat 8.3.5) was used to list all patient-related information, and patients diagnosed with stage I–II (node-negative) rectal cancer between January 1, 2004 and December 31, 2015 were identified from the SEER database (Figure 1). Rectal cancer patients were identified by the ICD-O-3 site codes C199 and C209 and behavior code 3 (NAACCR Items 522 and 523) (9). We chose to include these years because the information of preoperative serum CEA was recorded starting from 2004 and SEER follow-up ended in 2015.

Then, those received neoadjuvant radiotherapy, lack of positive histological confirmation, with unknown race, non-adenocarcinoma histologies, or not active follow-up were excluded from our study. Patients with preoperative serum CEA level available were included in our study, and we grouped “positive/elevated” and “negative/normal; within normal limits” as C1 and C0 (C-stage information). The cutoff values of CEA were 2.5 ng/ml for non-smokers and 5 ng/ml for smokers, respectively. In addition, the following patient clinicopathological variables were also retrieved from the SEER database: T stage, race, gender, tumor location, age at diagnosis, year of diagnosis, grade, and histology.

### Statistical Analysis

In the present study, Pearson's chi-squared test was used to compare all the patients' clinicopathological variables retrieved from the SEER database between the normal and elevated preoperative serum CEA levels. Some multivariable Cox proportional hazard models were built to identify independent prognostic variables of rectal cancer in our analyses. Survival curves were generated using Kaplan–Meier method, and differences between the curves were analyzed by log-rank test. The primary outcomes of interest in the present study were CSS and overall survival (OS). CSS was calculated from the date of diagnosis to the date of cancer-specific death. Deaths attributed to the rectal cancer were treated as events, and deaths from other causes were treated as censored observations at the date of death. To determine whether there was a significant interaction between the preoperative serum CEA level and T stage in predicting CSS, we also defined an interaction variable (T stage and serum CEA level). Two-sided *P* < 0.05 was considered statistically significant. Statistical analyses were mainly performed using the SPSS version 22 (IBM Corporation, Armonk, NY, USA).

## Results

### Patient Characteristics

Overall, 19,184 patients diagnosed with stage I–II rectal cancer patients were identified from the SEER database between January 1, 2004 and December 31, 2015 (Figure 1). Of these patients, 13,007 (67.8%) patients were assigned to C0 stage, and 6,177 (32.2%) patients were assigned to C1 stage. AJCC staging system suggested that 6,077 patients were in the T1 stage, 4,783 patients in the T2 stage, 7,021 in the T3 stage, and 1,303 were in the T4 stage. A total of 2,560 (13.3%) patients died of rectal cancer at the end of the follow-up time. The median follow-up time of the whole cohort was 44 months (0–143 months). Patients' baseline characteristics are summarized in Table 1.

**Table 1:** Comparison of baseline characteristics of stage I–II rectal cancer by the serum carcinoembryonic antigen (CEA) level.

**Variable**	**No. of patients (%)**		***P-*value**
	**CEA-normal**	**CEA-elevated**	
	**(*N* = 13,007)**	**(*N* = 6,177)**	
T stage			<0.001
T1	4,740 (36.4)	1,337 (21.6)	
T2	3,615 (27.8)	1,168 (18.9)	
T3	4,141 (31.8)	2,880 (46.6)	
T4	511 (3.9)	792 (12.8)	
Race			<0.001
White	10,800 (83.0)	4,833 (78.2)	
Black	999 (7.7)	702 (11.4)	
Other	1,208 (9.3)	642 (10.4)	
Gender			0.721
Male	7,475 (57.5)	3,533 (57.2)	
Female	5,532 (42.5)	2,644 (42.8)	
Tumor location			0.034
Rectosigmoid junction	4,378 (33.7)	2,175 (35.2)	
Rectum	8,629 (66.3)	4,002 (64.8)	
Age at diagnosis (years)			<0.001
≤65	6,392 (49.1)	2,482 (40.2)	
>65	6,615 (50.9)	3,695 (59.8)	
Year of diagnosis			0.254
2004–2009	6,697 (51.5)	3,126 (50.6)	
2010–2015	6,310 (48.5)	3,051 (49.4)	
Grade			<0.001
Grade I/II	10,872 (83.6)	5,007 (81.1)	
Grade III/IV	1,183 (9.1)	608 (9.8)	
Unknown	952 (7.3)	562 (9.1)	
Histology			<0.001
Adenocarcinoma	12,660 (97.3)	5,852 (94.7)	
Mucinous adenocarcinoma/	347 (2.7)	325 (5.3)	
Signet ring cell carcinoma			

### Improved Risk of T1 Stage Compared With Other T Stages in the Context of Serum CEA Elevation

It was found that C1 stage was more likely to correlate with higher T stage, black, rectosigmoid junction, older age, higher grade, and mucinous adenocarcinoma/Signet ring cell carcinoma (Table 1, *P* < 0.05). As shown in Table 2, race, gender, tumor location, age at diagnosis, year of diagnosis, grade, histology, T stage, and serum CEA level were included in the multivariate Cox analysis. When multivariate Cox analysis was performed, we convinced the following clinicopathological characteristics as independent prognostic factors in stage I–II rectal cancer; these included race, gender, tumor location, age at diagnosis, tumor grade, T stage, and serum CEA level, while the risk between T2,C0 and T1,C0 was not statistical difference (*P* = 0.925). It was shown that, in the context of serum CEA elevation, T1 stage presented unexpected higher risk of rectal-cancer-specific mortality compared with stages T2 and T3. In stage T1 disease, elevated level of serum CEA was associated with 227.6% increased risk of mortality compared to normal level of serum CEA.

**Table 2:** Multivariate Cox regression analyses of cause-specific survival (CSS) in stage I–II rectal cancer.

**Variable**	**University analysis**		**Multivariate analysis**	
	**HR (95% CI)**	***P-*value**	**HR (95% CI)**	***P-*value**
Race		<0.001		<0.001
White	Reference		Reference	
Black	1.453 (1.288–1.638)	<0.001	1.316 (1.166–1.485)	<0.001
Other	0.824 (0.712–0.952)	0.009	0.803 (0.694–0.928)	0.003
Gender		0.025		0.001
Male	Reference		Reference	
Female	0.914 (0.845–0.989)		0.874 (0.808–0.946)	
Tumor location		<0.001		<0.001
Rectosigmoid junction	Reference		Reference	
Rectum	1.402 (1.287–1.526)		1.541 (1.412–1.681)	
Age at diagnosis (years)		<0.001		<0.001
≤65	Reference		Reference	
>65	1.943 (1.791–2.107)		1.908 (1.758–1.681)	
Year of diagnosis		0.209		0.702
2004–2009	Reference		Reference	
2010–2015	1.057 (0.969–1.153)		1.017 (0.932–1.110)	
Grade		<0.001		<0.001
Grade I/II	Reference		Reference	
Grade III/IV	1.320 (1.166–1.494)	<0.001	1.187 (1.047–1.344)	0.007
Unknown	2.058 (1.827–2.318)	<0.001	1.815 (1.606–2.052)	<0.001
Histology		<0.001		0.272
Adenocarcinoma	Reference		Reference	
Mucinous adenocarcinoma/Signet ring cell carcinoma	1.504 (1.256–1.802)		0.902 (0.751–1.084)	
T stage and serum carcinoembryonic antigen level		<0.001		<0.001
T1, C0	Reference		Reference	
T1, C1	3.656 (3.106–4.303)		3.276 (2.781–3.858)	<0.001
T2, C0	0.960 (0.815–1.131)		1.008 (0.855–1.188)	0.925
T2, C1	1.707 (1.393–2.090)		1.694 (1.382–2.077)	<0.001
T3, C0	1.987 (1.734–2.277)		2.142 (1.867–2.457)	<0.001
T3, C1	3.336 (2.912–3.821)		3.535 (3.080–4.056)	<0.001
T4, C0	5.263 (4.318–6.414)		5.749 (4.712–7.015)	<0.001
T4, C1	9.957 (8.542–11.608)		10.404 (8.901–12.160)	<0.001

**Figure 2:**
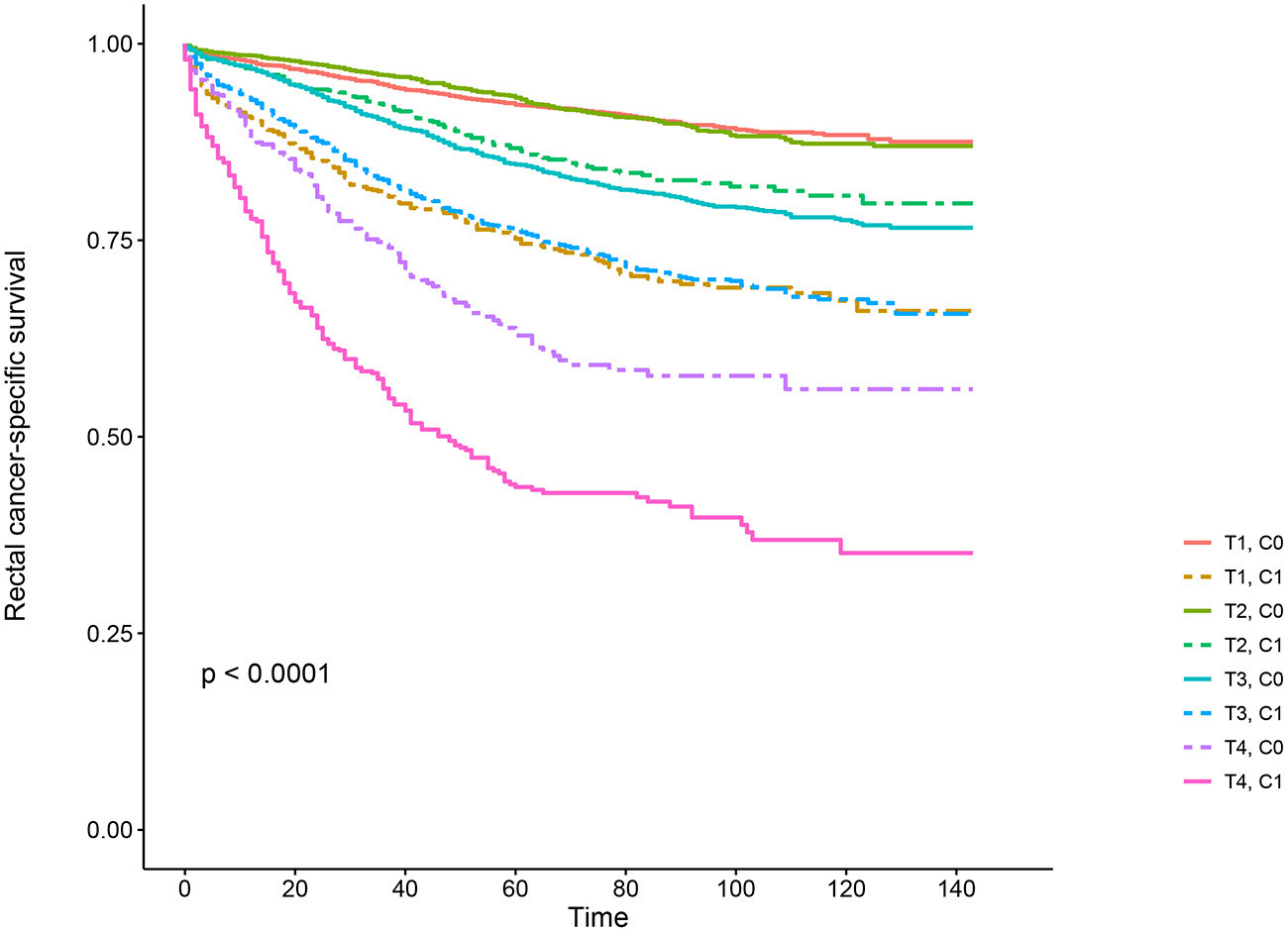
Kaplan–Meier cause-specific survival (CSS) curves of T stage combined with the serum carcinoembryonic antigen (CEA) level.

**Figure 3:**
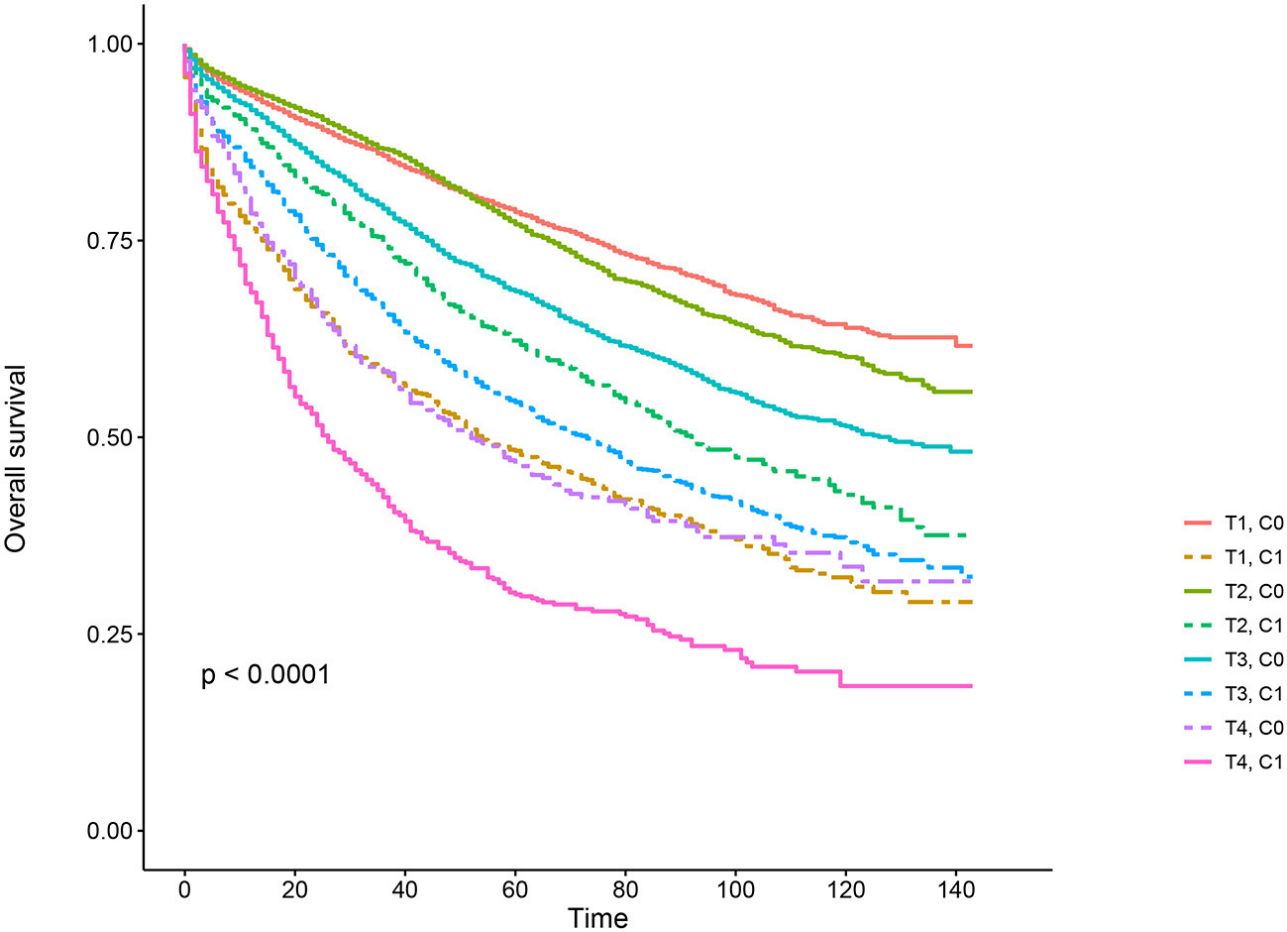
Kaplan–Meier overall survival (OS) curves of T stage combined with the serum carcinoembryonic antigen (CEA) level.

Kaplan–Meier survival curves are plotted in Figures 2, 3. The 5-year CSS rate was 92.2% in T1C0, 75.2% in T1C1, 93.1% in T2C0, 86.7% in T2C1, 84.6% in T3C0, 76.3% in T3C1, 62.9% in T4C0, and 43.6% in T4C1 (Figure 2, *P* < 0.001). Therefore, T1C1 presented a similar 5-year CSS rate compared with T3C1 (75.2 vs. 76.3%, *P* = 0.238). In addition, the finding was even more pronounced in OS. The 5-year OS rate was 78.6% in T1C0, 48.3% in T1C1, 77.1% in T2C0, 62.3% in T2C1, 68.5% in T3C0, 54.5% in T3C1, 45.3% in T4C0, and 30.0% in T4C1 (Figure 3, *P* < 0.001). T1C1 presented significantly lower 5-year CSS rate compared with T3C1 (48.3 vs. 54.5%, *P* < 0.001).

### Effect of Preoperative Serum CEA Elevation in T Stage

**Figure 4:**
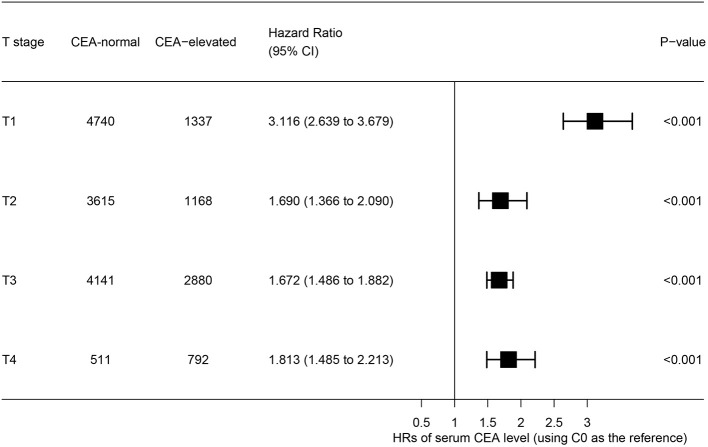
Hazard ratios (HRs) of cause-specific survival (CSS) between normal and elevated level of serum carcinoembryonic antigen (CEA) according to the T stage.

The forest plot was drawn to show the hazard ratios (HRs) to compare CSS between normal- and elevated-CEA groups in respective T stages (Figure 4). Compared with normal level of serum CEA, in stage T2, elevated level of serum CEA was associated with 69.0% increased risk of rectal-cancer-specific mortality [HR = 1.690, 95% confidence interval (CI) = 1.366–2.090, *P* < 0.001]; in stage T3, elevated level of serum CEA was associated with 67.2% increased risk of rectal-cancer-specific mortality (HR = 1.672, 95% CI = 1.486–1.882, *P* < 0.001); in stage T4, elevated level of serum CEA was associated with 81.3% increased risk of rectal-cancer-specific mortality (HR = 1.813, 95% CI = 1.485–2.213, *P* < 0.001); by contrast, however, in stage T1, elevated level of serum CEA presented even up to 211.6% increased risk of rectal-cancer-specific mortality (HR = 3.116, 95% CI = 2.639–3.679, *P* < 0.001).

## Discussion

We aimed to investigate the association of T stage and serum CEA levels in determining CSS of rectal cancer. We hypothesized that rectal cancer with very early T stage (stage T1) and serum CEA elevation may be a biological surrogate for aggressive disease, thus predicting a poor oncological outcome. With ~20,000 patients diagnosed with stage I–II rectal cancer included in our study.

In this study, it was found that serum CEA elevation was more likely to correlate with higher T stage, black, rectosigmoid junction, older age, and higher grade and mucinous adenocarcinoma/Signet ring cell carcinoma. Kaplan–Meier survival analyses showed that, in the context of serum CEA elevation, 5-year CSS rate of stage T1 was 75.2%, which did not achieve statistical difference from stage T3 (76.3%). When it comes to overall survival, 5-year OS rate of stage T1 with preoperative serum CEA elevation (48.3%) was even lower than stage T3 involved in CEA elevation (54.5%) and did not achieve statistical difference from stage T4 (45.3%), meaning elevated preoperative serum level of CEA could identify a subgroup of stage T1 rectal cancers with similar CSS compared with some stage T3 diseases and with similar OS compared with some stage T4 diseases.

After adjusting for known rectal cancer prognostic factors (race, gender, tumor location, age at diagnosis, year of diagnosis, grade, and histology), the interaction variable (T stage and serum CEA level) we defined was demonstrated to be an independent prognostic factor of rectal cancer. Multivariate Cox analysis showed that, in the context of serum CEA elevation, T1 stage presented unexpected higher risk of rectal-cancer-specific mortality compared with stages T2 and T3. In stage T1 disease, elevated level of serum CEA was associated with 227.6% increased risk of mortality compared to normal level of serum CEA. In addition, race, gender, tumor location, age at diagnosis, and tumor grade were also identified as independent prognostic factors of stage I–II rectal cancer. Apart from T1, it was found that preoperative serum CEA elevation presented ~75% higher risks of rectal-cancer-specific mortality in respective T stage, yet the number greatly increased to 211.6% in T1 stage.

In 2000, the Colorectal Working Group of the AJCC proposed the inclusion of serum level of CEA (C stage) into the conventional AJCC TNM staging system of colorectal cancer (18). Moreover, the American Society of Clinical Oncology and the European Group on Tumor Markers have both supported the inclusion of preoperative serum CEA level as a prognostic tool in colorectal cancer (19–21).

Several previous researches reported the serum CEA level as a strong prognostic role in colon cancer (4–7, 22–26). In 2011, Thirunavukarasu et al. (25) reported that preoperative serum CEA level was an independent prognostic biomarker of colon cancer, and prognosis was worse in high CEA patients with a lower stage compared with low CEA patients with a higher stage. In that study, high CEA was even deemed as strong as node positivity for predicting poor oncologic outcomes of colon cancer.

Yet, few studies focus on elucidating the prognostic role of serum CEA level in rectal cancer. In 2016, also using the SEER database, Tarantino et al. (9, 27) conducted the two large population-based investigations, which provided compelling evidence that elevated level of preoperative serum CEA was a strong predictor of worse overall and cancer-specific survival in rectal cancer. In 2018, Liu et al. (8) demonstrated that preoperative serum CEA was an independent prognostic factor of rectal cancer, and elevated serum CEA level presented evidently poorer survival compared with normal serum CEA level in stages I–IV. However, the only two previous studies focused on investigating the prognostic role of preoperative serum CEA level did not examine the association of CEA level and T stage in predicting the tumor outcomes of rectal cancer.

Although the conventional shows that cancer acquires the metastatic potential step by step as they grow to a large size (28), however, some previous studies indicated that acquisition of metastatic potential could occur very early in tumor progression. A previous research demonstrated the extremely poor survival of very small tumor size when involved in lymph node positivity (17). In addition, findings of our research show that elevation of preoperative serum CEA level in very early stage (stage T1) rectal cancer is associated with very poor OS and CSS and may be a surrogate of aggressive biology. We believe that both lymph node involvement and serum CEA elevation are deemed as the acquisition of metastatic ability. Our study, combined with that of Wo et al. consistently support the aforementioned idea that acquisition of metastatic potential could occur very early in tumor progression, thus associated with poor oncologic outcomes, and the initial biological feature are more likely to determine the potential of distant metastasis during cancer progression, rather than the accumulated metastatic ability (29).

Moreover, findings of our study are of clinical significance. Currently, stage I rectal cancer is treated with radical surgical resection alone because of relatively favorable oncologic outcomes. However, approximately 10–15% of patients will develop tumor recurrence after radical resection (30, 31). In the present study, we happen to identify a subgroup of stage T1 rectal cancer with very poor prognosis compared with the rest of stage I rectal cancer, meaning that those stage T1 rectal cancer with involvement of preoperative serum CEA elevation should catch more attention of oncologists.

However, there are two limitations in our study. On the one hand, this study did not include some known prognostic factors of rectal cancer in our analyses, including microsatellite instability status and postoperative complications, which were not available from the SEER database and might introduce biases to some extent. We cannot provide ranges and mean values of CEA in both groups. On the other hand, the present study was retrospective rather than based on prospective data. Therefore, our findings still need to be validated in other cohorts, especially in large prospective clinical studies.

In conclusion, our study demonstrates that stage T1 rectal cancer, when involved in preoperative serum CEA elevation, may be a surrogate of biologically aggressive disease and correlate with unfavorable OS and CSS. Moreover, this subgroup of rectal cancer deserves more clinical attention of oncologists. Our findings, if validated in future database studies, would provide a new therapy idea for early stage rectal cancer.

## Data Availability Statement

The datasets generated for this study are available on request to the corresponding author.

## Ethics Statement

Patients in this study were identified from the SEER database, and the approval for use of the data was obtained through a request submitted to the SEER program. The approval by the institutional review board was not needed because SEER database is publicly available.

## Author Contributions

SW and WG have made a substantial and direct contribution to this study, and approved it for submission.

### Conflict of Interest

The authors declare that the research was conducted in the absence of any commercial or financial relationships that could be construed as a potential conflict of interest.
